# Molecular and immune correlates of *TIM*-*3* (*HAVCR2*) and galectin 9 (*LGALS9*) mRNA expression and DNA methylation in melanoma

**DOI:** 10.1186/s13148-019-0752-8

**Published:** 2019-11-20

**Authors:** Tobias A. W. Holderried, Luka de Vos, Emma Grace Bawden, Timo J. Vogt, Joern Dietrich, Romina Zarbl, Friedrich Bootz, Glen Kristiansen, Peter Brossart, Jennifer Landsberg, Dimo Dietrich

**Affiliations:** 1 Department of Oncology, Hematology and Rheumatology, University Hospital Bonn, Bonn, Germany; 2 Department of Otolaryngology, Head and Neck Surgery, University Hospital Bonn, Sigmund-Freud-Str. 25, 53105 Bonn, Germany; 3 Unit for RNA Biology, Department of Clinical Chemistry and Clinical Pharmacology, University Hospital Bonn, Bonn, Germany; 4 Institute of Experimental Oncology (IEO), University Hospital Bonn, Bonn, Germany; 5 Department of Microbiology & Immunology, The University of Melbourne at the Peter Doherty Institute for Infection & Immunity, Melbourne, VIC Australia; 6 Institute of Pathology, University Hospital Bonn, Bonn, Germany; 7 Department of Dermatology and Allergy, University Hospital Bonn, Bonn, Germany

**Keywords:** TIM-3, *HAVCR2*, Galectin 9, *LGALS9*, DNA methylation, Melanoma, Biomarker, Immunotherapy, Prognosis, Prediction

## Abstract

**Background:**

The T cell immunoglobulin and mucin-domain containing-3 receptor TIM-3 (also known as hepatitis A virus cellular receptor 2, encoded by *HAVCR2*) and its ligand galectin 9 (*LGALS9*) are promising targets for immune checkpoint inhibition immunotherapies. However, little is known about epigenetic regulation of the encoding genes. This study aimed to investigate the association of *TIM*-*3* and *LGALS9* DNA methylation with gene expression, patients’ survival, as well as molecular and immune correlates in malignant melanoma.

**Results:**

Methylation of all six *TIM*-*3* CpGs correlated significantly with TIM-3 mRNA levels (*P* ≤ 0.05). A strong inverse correlation (Spearman’s *ρ* = − 0.49) was found in promoter regions, while a strong positive correlation (*ρ* = 0.63) was present in the gene body of *TIM*-*3*. High TIM-3 mRNA expression (hazard ratio (HR) = 0.88, 95% confidence interval (CI) [0.81–0.97], *P* = 0.007) was significantly associated with better overall survival. Seven of the eight *LGALS9* CpG sites correlated significantly with LGALS9 mRNA levels (*P* ≤ 0.003). Methylation at five CpG sites showed a strong inverse correlation (Spearman’s *ρ* = − 0.67) and at two sites a weak positive correlation (Spearman’s *ρ* = 0.15). High LGALS9 mRNA expression was significantly associated with increased overall survival (HR = 0.83, 95%CI [0.75–0.93], *P* = 0.001). In addition, we found significant correlations between *TIM*-*3* and *LGALS9* methylation and mRNA expression with immune cell infiltrates and significant differences among distinct immune cell subsets.

**Conclusions:**

Our study points toward an epigenetic regulation of *TIM*-*3* and *LGALS9* via DNA methylation and might provide an avenue for the development of a predictive biomarker for response to immune checkpoint blockade.

## Introduction

Immunotherapy has revolutionized cancer treatment in recent years. One main principle of anti-cancer immunotherapy is immune checkpoint blockade (ICB), which was recognized with the Nobel Prize in 2018 [1]. Immune checkpoint pathways play a major role in tumor immune resistance, especially by evasion from cytotoxic T lymphocytes which are specific for tumor antigens [reviewed in 2]. Physiologically, T cells are controlled via immune checkpoint pathways in order to allow for self-tolerance and prevent destruction of normal tissues in the context of immune response. Interference with different inhibitory pathways enables tumor cells to avoid antigen-specific T cell reactions [2]. ICB refers, among others, to the inhibition of the interaction between tumor-infiltrating lymphocytes (TILs) and tumor cells, resulting in an augmented anti-tumor immune response [2].

Treatment of malignant melanoma, the most aggressive skin cancer, has been at the forefront of ICB [3]. As a result, ICB has become a standard treatment option for advanced melanoma which has led to a dramatic improvement in the prognosis of these patients [3–6]. Food and Drug Administration (FDA)-approved inhibitors target the immune checkpoints cytotoxic T-lymphocyte-associated protein 4 (CTLA-4), programmed cell death protein 1 (PD-1), and programmed cell death ligand 1 (PD-L1). However, various other antagonists and agonists targeting additional immune checkpoints are currently under clinical investigation, among them the T cell immunoglobulin and mucin-domain containing-3 (TIM-3) immune checkpoint.

TIM-3, encoded by the hepatitis A virus cellular receptor 2 gene (*HAVCR2*), is a trans-membrane receptor expressed by a wide range of cells including T lymphocytes, innate immune cells such as monocytes, natural killer (NK), and dendritic cells (DC), and additionally on cancer stem cells [7, 8]. One ligand of TIM-3 is the C-type lectin galectin 9 (LGALS9) [9]. Galectin 9, encoded by the gene *LGALS9*, is physiologically expressed in multiple cell types, especially in cells of lymphatic organs and in monocytes, but also in different tissue endothelial cells, small intestine, and in target cells for different viruses such as the hepatitis C virus [10–15]. Additionally, LGALS9 is expressed by tumor cells which can have diverse effects on different immune cells in the tumor microenvironment (reviewed in [16]). Expression of TIM-3 is notably associated with T cell exhaustion and impaired T cell function [7]. The interaction between TIM-3 and galectin 9 has been shown to induce apoptosis in effector T helper 1 (Th1) cells [17], consequently resulting in a reduction of autoimmune and anti-tumor immune responses [17–20]. This renders TIM-3 an attractive candidate target for ICB.

Reports on the epigenetic regulation of TIM-3 and LGALS9 in melanoma are sparse. Elucidating the regulation of TIM-3 and LGALS9 on an epigenetic level might help to understand the response and resistance mechanisms to TIM-3 ICB and consequently could be a prerequisite for the identification and development of mechanism-driven biomarkers for patient stratification, i.e., predictive biomarkers. Among epigenetic mechanisms, DNA methylation is of fundamental importance in multiple biological processes, including embryogenesis, imprinting, X chromosome inactivation, T cell differentiation (including T cell exhaustion), and tumorigenesis [21–24]. Promoter hypermethylation is frequently associated with transcriptionally silenced genes, while increased levels of gene body methylation are normally found in genes with high transcriptional activity [24]. In melanoma, DNA methylation has already been shown to have an important impact on gene transcription, and is believed to play a central role in pathogenesis and disease progression (reviewed in [25]). DNA demethylation of the *TIM*-*3* promoter in T cells has already been reported to be critical for stable expression [26]. Two recent studies identified *TIM*-*3* hypomethylation in colorectal and breast cancer tissues compared to normal tissues [27, 28].

Considering TIM-3 as a promising candidate for ICB, we analyzed the methylation status at single CpG site resolution as well as the corresponding mRNA levels of TIM-3 and its ligand LGALS9 in *N* = 470 melanoma patients provided by The Cancer Genome Atlas (TCGA) [29], and analyzed *TIM*-*3*/*LGALS9* methylation levels in isolated immune cells, melanocyte and melanoma cell lines.

## Results

### *HAVCR2* and *LGALS9* methylation correlates with TIM-3 and LGALS9 mRNA expression

**Fig. 1:**
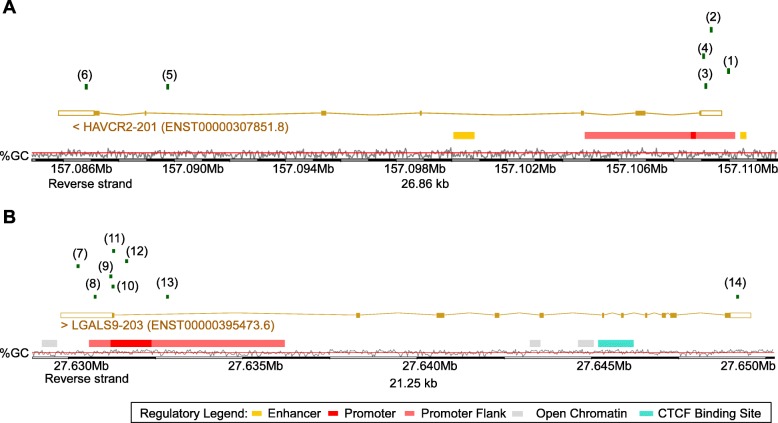
Genomic Organization of the *TIM*-*3* (*HAVCR2*) (**a**) and *LGALS9* (**b**) genes. Shown are CG-density and target sites of HumanMethylation450 BeadChip beads. The modified illustration was exported from www.ensemble.org (release 96) and is based on Genome Reference Consortium Human Build 38 patch release 12 (GRCh38.p12). Beads are numbered as follows: cg19110684 (1), cg19646897 (2), cg15371617 (3), cg17484237 (4), cg19063654 (5), cg18374914 (6) (all *TIM-3*); cg19654781 (7), cg10699049 (8), cg27625456 (9), cg21157094 (10), cg23290146 (11), cg05105919 (12), cg03909504 (13), cg06852032 (14) (all *LGALS9*)

A total of 14 CpG sites were investigated within the *HAVCR2* and *LGALS9* gene loci. Of those, CpGs targeted by beads cg19110684 (1), cg19646897 (2), cg15371617 (3), cg17484237 (4), cg19063654 (5), and cg18374914 (6) were used to assess methylation at the *HAVCR2* gene locus and beads cg19654781 (7), cg10699049 (8), cg27625456 (9), cg21157094 (10), cg23290146 (11), cg05105919 (12), cg03909504 (13), and cg06852032 (14) allowed for methylation analysis of the *LGALS9* gene (Fig. 1). While the beads 1–4 (*HAVCR2*) and 7–13 (*LGALS9*) target CpG sites within the predicted promoter regions, beads 5–6 (*HAVCR2*) and 14 (*LGALS9*) were located in the gene bodies.

**Fig. 2:**
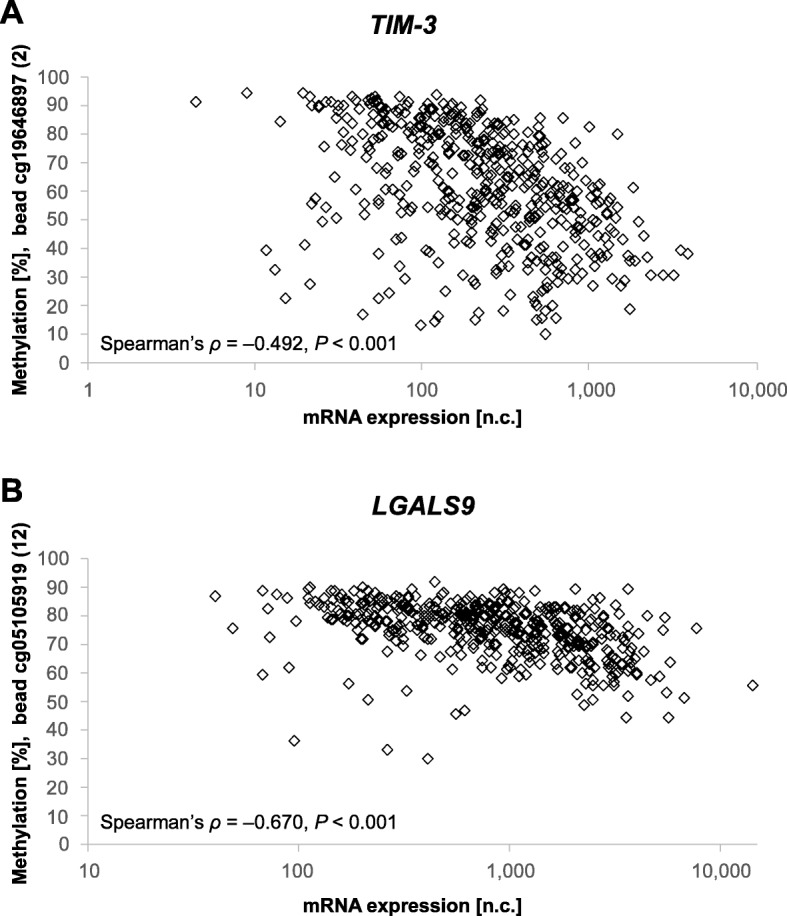
Correlations between *TIM*-*3* and *LGALS9* methylation with mRNA expression. Correlations between methylation of CpG site 2 (*TIM*-*3*, cg19646897, **a**) and CpG site 12 (*LGALS9*, cg05105919, **b**) with respective mRNA expression in *N* = 468 melanoma samples

Firstly, we investigated *HAVCR2* and *LGALS9* methylation with their respective mRNA expression (Table 1). For both genes, we observed characteristic methylation patterns shared by many epigenetically regulated genes, that is, low methylation levels in the central promoter region, increasing methylation levels of the promoter flanks, and high methylation levels within the gene body. In accordance, we found significant inverse correlations between mRNA expression and methylation in the promoter region (beads 1–4 and 7–12) while a significant and strong positive correlation was present in the gene body (beads 5, 6, and 14) and the more intragenic located promoter region (bead 13). Representative graphs (beads 2 and 12) of the correlation of mRNA expression and methylation are shown in Fig. 2. CpG site targeted by bead 8 was the only site which did not reach statistical significance. The strongest correlations, as indicated by Spearman’s *ρ* > 0.6 and *ρ* < − 0.6, were present within the intragenic 3′-UTR of *HAVCR2* (bead 6) and the central promoter region of *LGALS9* (bead 12).

**Table 1:** Correlations of *TIM-3* and *LGALS9* methylation with TIM-3 and galectin 9 mRNA expression, lymphocyte score and overall survival

Analyte	Mean methylation [%]/mRNA expression [n.c.]; [95% CI]	Correlation with mRNA expression		Correlation with lymphocyte score		Overall survival (Cox proportional hazards analysis)	
		Spearman’s *ρ*	*P* value	Spearman’s *ρ*	*P* value	Hazard ratio [95% CI]	*P* value
TIM-3 mRNA	412; [367–457]	NA	NA	**0.506**	**< 0.001**	**0.88 [0.81–0.97]**	**0.007**
*TIM-3* cg19110684 (1)	50.1; [47.6–52.6]	**− 0**.**408**	**< 0.001**	**− 0.272**	**< 0.001**	1.07 [0.93–1.25]	0.35
*TIM-3* cg19646897 (2)	61.4; [59.5–63.2]	− **0**.**492**	< **0**.**001**	− **0**.**315**	< **0**.**001**	1.20 [0.89–1.63]	0.24
*TIM-3* cg15371617 (3)	13.2; [12.1–14.3]	− **0**.**189**	< **0**.**001**	− **0**.**118**	**0**.**033**	1.04 [0.88–1.24]	0.62
*TIM-3* cg17484237 (4)	19.7; [18.4–21.0]	− **0**.**114**	**0**.**013**	− 0.071	0.20	1.08 [0.91–1.27]	0.37
*TIM-3* cg19063654 (5)	57.9; [55.7–60.2]	**0**.**263**	< **0**.**001**	**0**.**179**	**0**.**001**	0.91 [0.75–1.10]	0.35
*TIM-3* cg18374914 (6)	35.9;[33.4–36.6]	**0**.**633**	< **0**.**001**	**0**.**432**	< **0**.**001**	0.87 [0.74–1.04]	0.12
Galectin 9 mRNA	1306;[1183-1429]	NA	NA	**0**.**352**	**< 0.001**	**0**.**83** [**0**.**75**–**0.93**]	**0**.**001**
*LGALS9* cg19654781 (7)	68.2; [67.5–69.0]	− **0**.**186**	< **0**.**001**	− **0**.**135**	**0**.**014**	0.87 [0.35–2.18]	0.77
*LGALS9* cg10699049 (8)	79.2; [78.4–80.0]	− 0.045	0.33	− **0**.**128**	**0**.**020**	0.80 [0.33–1.96]	0.63
*LGALS9* cg27625456 (9)	17.4; [15.7–19.0]	− **0**.**404**	< **0**.**001**	− 0.034	0.54	1.02 [0.91–1.14]	0.77
*LGALS9* cg21157094 (10)	22.3; [21.0–23.6]	− **0**.**371**	< **0**.**001**	−0.091	0.10	1.07 [0.92–1.24]	0.41
*LGALS9* cg23290146 (11)	33.6; [32.2–35.0]	− **0**.**418**	**< 0**.**001**	**−0.188**	**0**.**001**	1.23 [0.98–1.54]	0.078
*LGALS9* cg05105919 (12)	50.8; [49.5–52.1]	− **0**.**670**	< **0**.**001**	− **0**.**351**	< **0**.**001**	1.30 [0.93–1.81]	0.12
*LGALS9* cg03909504 (13)	31.9; [30.7–33.0]	**0**.**135**	**0**.**003**	**0**.**196**	< **0**.**001**	0.84 [0.66–1.06]	0.14
*LGALS9* cg06852032 (14)	85.7; [84.8–86.6]	**0**.**146**	**0**.**001**	0.014	0.80	1.32 [0.58–3.02]	0.50

*TIM*-*3* and *LGALS9* methylation was determined at 14 different loci targeted by HumanMethylation450 BeadChip beads (Fig. 1) in *N* = 470 melanoma patients from The Cancer Genome Atlas. Significant features are shown in boldface.

*NA* not applicable

### *HAVCR2* and *LGALS9* methylation is correlated to clinical-pathological, molecular, and immunologic features

Secondly, we examined correlations between mRNA expression/gene methylation of *TIM*-*3* and *LGALS9* and clinical-pathological and molecular features. For TIM-3, we found positive correlations between mRNA expression and staging parameters such as T-category, AJCC7 stage, and Clark level. Conversely, we found negative correlations between mRNA expression and Breslow depth. However, we did not observe significant correlations between the aforementioned staging parameters and methylation of any analyzed CpG sites of *HAVCR2*. In contrast, for *LGASL9*, methylation of particular CpG sites correlated positively with all aforementioned parameters. LGASL9 mRNA expression correlated positively with AJCC7 stage and T-category and correlated inversely with Breslow depth.

We investigated molecular data published by the TCGA Research Network [29] (Additional file 1: Table S1). Since the TIM-3/galectin 9 axis is strongly involved in T cell exhaustion [7], we investigated potential correlations of mRNA expression of both genes and expression of the T cell exhaustion markers PD-1 and the lymphocyte-activation gene 3 (LAG-3). Indeed, we found strong positive correlations between mRNA expression of TIM-3 and PD-1 (*ρ* = 0.830), TIM-3 and LAG-3 (*ρ* = 0.844), as well as between LGALS9 and PD-1 (*ρ* = 0.755), and LGLAS9 and LAG-3 (*ρ* = 0.753) (all *P* < 0.001). Based on genomic analyses of the most prevalent mutations in the tumors (significantly mutated genes), the TCGA Research Network classified four molecular subtypes for melanoma patients: mutant *BRAF*, mutant *RAS*, mutant *NF1*, and Triple-Wildtype [29]. Relating to that finding, we examined whether DNA methylation differed between the aforementioned molecular subgroups. Indeed, we found *HAVCR2* methylation correlating positively with mutational subtype (beads 2–5) and in particular with *BRAF* mutation (beads 1–5), with lower methylation levels present in *BRAF*-mutated compared to -wildtype tumors (see Additional file 1). However, such correlations were not present within the *LGALS9* gene (see Additional file 1). Thirdly, we investigated the relationship between *HAVCR2*/*LGALS9* and immunologic features such as lymphocyte infiltration and immune stimulating interferon-γ (IFN-γ) signature. To estimate the lymphocyte infiltration, we used the lymphocyte score, a semi-quantitative method to assess the number of lymphocytes in a sample. Similar to the relationship we observed between methylation and mRNA expression, methylation status in the promoter regions correlated inversely with lymphocyte score while methylation in the gene body (beads 5–6) and the intragenic promoter region (bead 13) showed a positive correlation. Accordingly, mRNA expression of both TIM-3 and LGALS9 correlated strongly and positively with the lymphocyte score (Table 1). Furthermore, we found a significant positive correlation between tumor purity and promoter methylation (*HAVCR2*: beads 1–2, *LGALS9*: beads 7–12) and inverse correlations between tumor purity and mRNA expression as well as gene body methylation (*HAVCR2*: beads 5–6, *LGALS9*: bead 13) (see Additional file 1). In our prior analyses, methylation levels correlated strongly with immune infiltration and differed depending on *BRAF* status. Since Thorsson et al. found driver mutations, such as *BRAF*, correlated positively with higher leukocyte levels [30], we investigated potential differences in immune infiltration with regard to *BRAF* status. Hence, we tested the leukocyte fraction and tumor purity in *BRAF*-mutated and *BRAF*-wildtype melanoma. We observed statistically significant differences between *BRAF*-mutated and -wildtype tumors and tumor purity (*P* = 0.036), but no statistically significant results for the leukocyte fraction (*P* = 0.057).

The expression of TIM-3 and LGALS9 is thought to be at least partly regulated through interferon-γ and is expressed on IFN-γ-producing cells [7]. Therefore, correlation analyses were performed between the mRNA expression/methylation levels of TIM-3 and LGALS9 and an INF-γ signature. We used INF-γ expression as well as expression levels of INF-γ-regulated genes (*STAT1*, *STAT2*, *JAK2*, and *IRF9*) as surrogate for an INF-γ signature (Table 2). We found strong, positive correlations with mRNA expression of TIM-3 and LGALS9 and an IFN-γ signature. DNA methylation analysis of *HAVCR2* and *LGALS9* showed the same correlation pattern as observed with mRNA expression and lymphocyte score; methylation status in the promoter regions correlated inversely with the INF-γ signature while methylation in the gene body showed a positive correlation.

**Table 2:** Correlations of *TIM-3* and *LGALS9* methylation and TIM-3 and galectin 9 mRNA expression with interferon-γ signature

Analyte	IFN-γ		STAT1		STAT2		JAK2		IRF9	
	Spearman’s *ρ*	*P* value	Spearman’s *ρ*	*P* value	Spearman’s *ρ*	*P* value	Spearman’s *ρ*	*P* value	Spearman’s *ρ*	*P* value
TIM-3 mRNA	**0**.**795**	< **0**.**001**	**0**.**694**	< **0**.**001**	**0**.**297**	< **0**.**001**	**0**.**481**	< **0**.**001**	**0**.**525**	< **0**.**001**
*TIM-3* cg19110684 (1)	− **0**.**400**	< **0**.**001**	− **0**.**297**	< **0**.**001**	− **0**.**205**	< **0**.**001**	− **0**.**123**	**0**.**008**	− **0**.**323**	< **0**.**001**
*TIM-3* cg19646897 (2)	− **0**.**505**	< **0**.**001**	− **0**.**422**	< **0**.**001**	− **0**.**221**	< **0**.**001**	− **0**.**230**	< **0**.**001**	− **0**.**335**	< **0**.**001**
*TIM-3* cg15371617 (3)	− **0**.**254**	< **0**.**001**	− **0**.**224**	< **0**.**001**	− **0**.**191**	< **0**.**001**	− 0.052	0.26	− **0**.**206**	< **0**.**001**
*TIM-3* cg17484237 (4)	− **0**.**171**	< **0**.**001**	− **0**.**161**	< **0**.**001**	− **0**.**207**	< **0**.**001**	0.041	0.38	− **0**.**171**	< **0**.**001**
*TIM-3* cg19063654 (5)	**0.140**	**0.002**	**0.098**	**0.034**	− 0.036	0.44	**0.236**	**< 0.001**	0.062	0.18
*TIM-3* cg18374914 (5)	**0.549**	**< 0.001**	**0.417**	**< 0.001**	**0.201**	**< 0.001**	**0.340**	**< 0.001**	**0.397**	**< 0.001**
Galectin 9 mRNA	**0.639**	**< 0.001**	**0.556**	**< 0.001**	**0.322**	**< 0.001**	**0.346**	**< 0.001**	**0.613**	**< 0.001**
*LGALS9* cg19654781 (6)	**− 0.188**	**< 0.001**	**− 0.143**	**0.002**	− 0.052	0.26	0.001	0.99	**− 0.185**	**< 0.001**
*LGALS9* cg10699049 (7)	− 0.049	0.29	− 0.057	0.22	− 0.029	0.53	− 0.013	0.79	− 0.057	0.22
*LGALS9* cg27625456 (8)	**− 0.241**	**< 0.001**	**− 0.217**	**< 0.001**	**− 0.198**	**< 0.001**	− 0.067	0.15	**− 0.269**	**< 0.001**
*LGALS9* cg21157094 (9)	**− 0.282**	**< 0.001**	**− 0.252**	**< 0.001**	**− 0.201**	**< 0.001**	− 0.054	0.25	**− 0.270**	**< 0.001**
*LGALS9* cg23290146 (10)	**− 0.355**	**< 0.001**	**− 0.307**	**< 0.001**	**− 0.155**	**0.001**	**− 0.158**	**0.001**	**− 0.278**	**< 0.001**
*LGALS9* cg05105919 (11)	**− 0.536**	**< 0.001**	**− 0.426**	**< 0.001**	**− 0.231**	**< 0.001**	**− 0.299**	**< 0.001**	**− 0.397**	**< 0.001**
*LGALS9* cg03909504 (12)	**0.280**	**< 0.001**	**0.258**	**< 0.001**	**0.180**	**< 0.001**	**0.254**	**< 0.001**	**0.223**	**< 0.001**
*LGALS9* cg06852032 (13)	0.054	0.25	0.084	0.069	**0.105**	**0.022**	**0.161**	**< 0.001**	0.060	0.19

mRNA expression of IFN-γ, STAT1, STAT2, JAK2, and IRF9 is used as surrogate for an interferon-γ signature. Methylation and mRNA expression data were procurable from *N* = 468 patient samples. Significant features are shown in boldface

### *HAVCR2* and *LGALS9* are differentially methylated among melanocytes, melanoma cells, and leukocytes

**Fig. 3:**
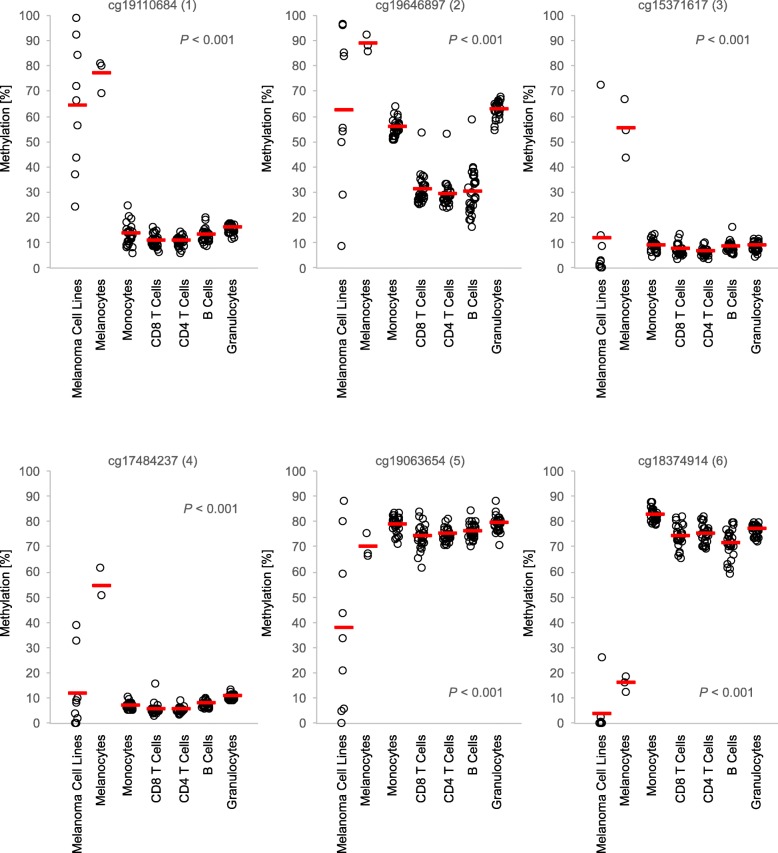
*TIM*-*3* methylation in leukocytes, melanocytes, and melanoma cell lines. *TIM*-*3* methylation at six sites in isolated leukocytes (monocytes, granulocytes, B cells, CD8^+^ T cells, and CD4^+^ T cells) from healthy donors (*N* = 28), melanocytes (*N* = 3), and melanoma cell lines (*N* = 9), extracted from the Geo Database. Red bars indicate mean methylation values. *P*-values refer to the Kruskal–Wallis test

**Fig. 4:**
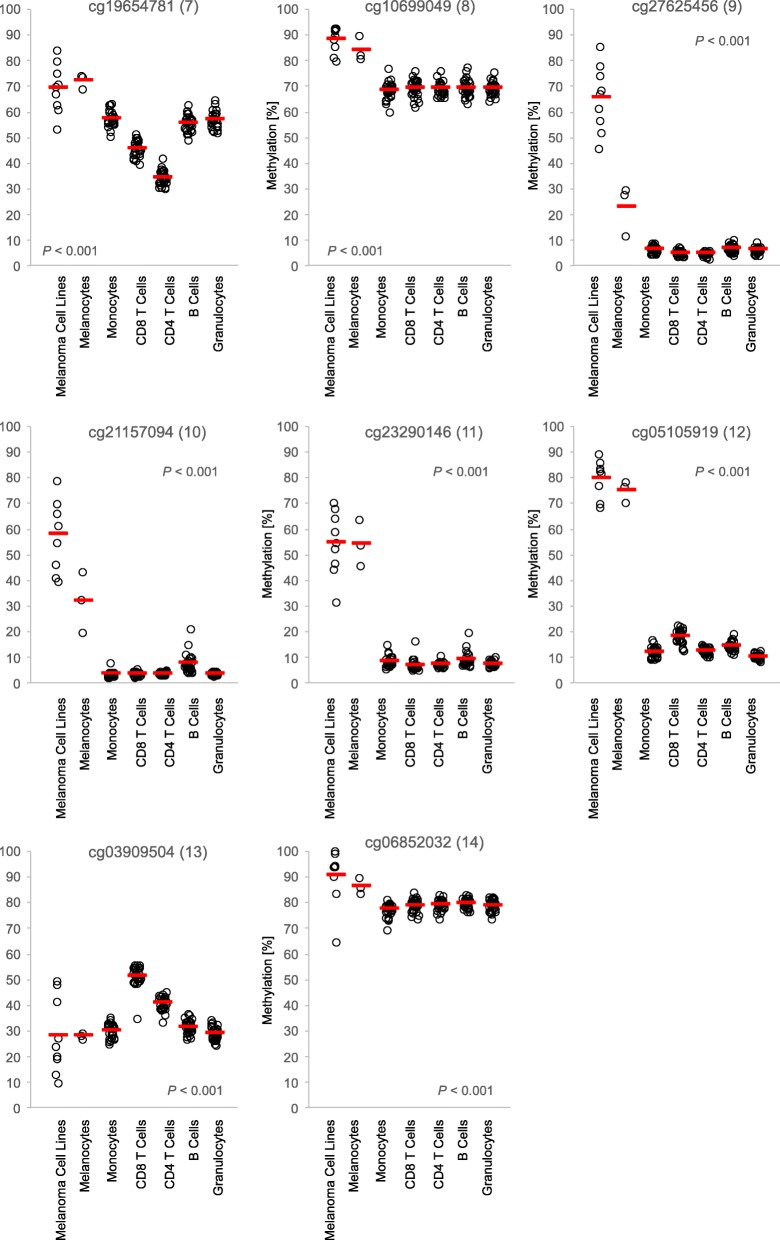
*LGALS9* methylation in leukocytes, melanocytes, and melanoma cell lines. *LGALS9* methylation at eight sites in isolated leukocytes (monocytes, granulocytes, B cells, CD8^+^ T cells, CD4^+^ T cells) from healthy donors (*N* = 28), melanocytes (*N* = 3) and melanoma cell lines (*N* = 9), extracted from the Geo Database. Red bars indicate mean methylation values. *P* values refer to the Kruskal–Wallis test

We next investigated methylation levels of *HAVCR2* and *LGALS9* in pure cell populations. We analyzed melanocyte and melanoma cell lines as well as immune cells, including monocytes, CD8^+^ and CD4^+^ T cells, B cells, and granulocytes. The immune cells were isolated from peripheral blood of healthy individuals. We observed distinct differences in methylation state between the different cell types and between different CpG sites (Figs. 3 and 4). At CpG sites 1–4, there was a substantial increase in *HAVCR2* methylation in melanocytes compared to leukocytes. Leukocytes showed overall low methylation levels at CpG sites located in the promoter of *HAVCR2* (beads 1, 3–4) and high levels in the gene body (beads 5–6). Similar to leukocytes, methylation levels in melanoma cell lines were low at CpG sites targeted by beads 3–4, but, in contrast, considerably higher at CpG site probed by bead 1. The greatest difference in methylation levels between leukocytes and melanoma cell lines within *HAVCR2* was found at CpG site targeted by bead 6. Furthermore, we observed variability within the groups, especially for melanoma cell lines which showed a particularly high variance. Interestingly, methylation levels were significantly different between different subsets of leukocytes, e.g., at bead target site two, lymphocytes (CD8^+^ T, CD4^+^ T, and B cells) had lower methylation levels compared to monocytes and granulocytes (Fig. 3**)**.

We made similar observations for *LGALS9*. In the promoter area (beads 9–12), leukocytes showed low methylation levels and melanoma and melanocyte cell lines were highly methylated. The highest difference was detected at CpG targeted by bead 12. Of note, CpG site 13 showed increased *LGALS9* methylation in CD8^+^ T cells not only compared to melanoma and melanocyte cell lines but also compared to distinct leukocytes including the CD4^+^ T lymphocytes (Fig. 4).

### *TIM-3* and *LGALS9* methylation and expression correlates to tumor infiltrating leukocytes

**Fig. 5:**
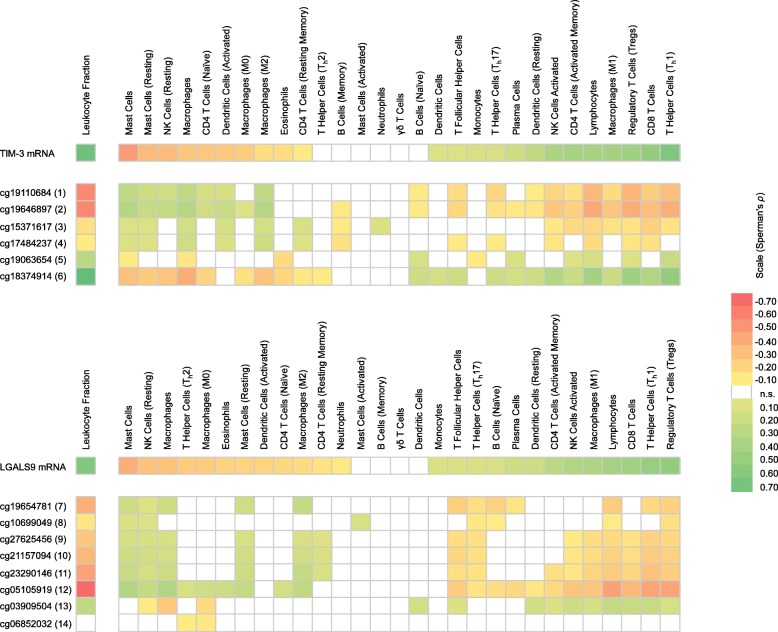
Correlations of *TIM*-*3* and *LGALS9* methylation and mRNA expression with leukocytes infiltration*.* Shown are Spearman’s rank correlations (Spearman’s *ρ*) between methylation/mRNA expression of TIM-3/LGALS9 and leukocyte fraction, as well as tumor infiltrating leukocytes, including lymphocytes (CD8^+^ T cells, regulatory T cells, T follicular helper cells, T helper cells 1, T helper cells 2, T helper cells 17, γδ T cells, naïve CD4^+^ T cells, resting and activated memory CD4^+^ T cells, naïve B cells, memory B cells, plasma cells, resting and activated natural killer cells), monocytes and macrophages (M0/M1/M2 macrophages), resting and activated dendritic cells, resting and activated mast cells, eosinophils, and neutrophils. Immune signatures of tumor infiltrating leukocytes were based on RNAseq analysis and the leukocyte fraction was based on methylation analysis. Only statistically significant (*P* < 0.05) are shown in color. *P* values and Spearman’s *ρ* correlation coefficients can be found in Additional file 1

We investigated correlations between methylation/mRNA expression of both genes and immune cells in the tumor microenvironment. Tumor-infiltrating leukocytes were evaluated via RNAseq immune cell signatures as surrogate for different cell types as described by Thorsson et al. [30] and via the leukocyte fraction assessed by DNA methylation arrays previously published by Saltz et al. [31] (Fig. 5, Additional file 1: Table S1). We found strong, significantly positive correlations between TIM-3 mRNA expression and leukocyte fraction (Spearman’s *ρ* = 0.758, *P* < 0.001), Th1 cells (*ρ* = 0.657, *P* < 0.001), CD8^+^ T cells (*ρ* = 0.527, *P* < 0.001), regulatory T cells (*ρ* = 0.465, *P* < 0.001), and total lymphocytes (*ρ* = 0.428, *P* < 0.001). Both *HAVCR2*/*TIM*-*3* gene body and promoter methylation correlated negatively with leukocyte fraction (strongest correlation found for gene body bead 6: *ρ* = − 0.819; and promoter bead 2: *ρ* = − 0.561, both *P* < 0.001) and CD8^+^ T cells (bead 6: *ρ* = − 0.354, bead 2: *ρ* = − 0.308, both *P* < 0.001). Furthermore, *HAVCR2*/*TIM*-*3* gene body methylation correlated positively and promoter methylation inversely with total lymphocytes (bead 6: *ρ* = 0.438, bead 2: *ρ* = − 0.408, both *P* < 0.001), Th1 cells (bead 6: *ρ* = 0.233, bead 2: *ρ* = − 0.380, both *P* < 0.001;), activated CD4^+^ memory T cells (bead 6: *ρ* = 0.220; bead 2: *ρ* = − 0.225, *P* < 0.001), and M1 macrophages (bead 6: *ρ* = 0.211, bead 2: *ρ* = − 0.277, both *P* < 0.001). We observed negative correlations between mRNA expression of TIM-3 and infiltration of mast cells (*ρ* = − 0.466, *P* < 0.001), macrophages (*ρ* = − 0.272, *P* = 0.001), and naïve CD4^+^ T cells (*ρ* = − 0.265, *P* < 0.001) (Fig. 5). Accordingly, we detected positive correlations of promoter methylation and negative correlations of gene body methylation with infiltration of the aforementioned cell types. We obtained similar results for the correlation analyses with LGALS9, whereby mRNA expression positively correlated with leukocyte fraction (Spearman’s *ρ* = 0.646, *P* < 0.001), regulatory T cells (*ρ* = 0.533, *P* < 0.001), Th1 cells (*ρ* = 0.509, *P* < 0.001), CD8^+^ T cells (*ρ* = 0.456, *P* < 0.001), total lymphocytes (*ρ* = 0.429, *P* < 0.001), M1 macrophages (*ρ* = 0.378, *P* < 0.001), and activated NK cells (*ρ* = 0.348, *P* < 0.001). Depending on the position of the CpG sites, we detected significant positive and negative correlations between methylation and aforementioned leukocyte infiltrates. Strongest negative correlations between LGALS9 mRNA expression were found for total mast cells (*ρ* = − 0.382, *P* < 0.001), resting NK cells (*ρ* = − 0.288, *P* < 0.001), M0 macrophages (*ρ* = − 0.244, *P* < 0.001), and eosinophils (*ρ* = − 0.210, *P* < 0.001), accompanied by similar correlation patterns with methylation levels as seen before, with positive correlations for methylation at CpG sites representing the promoter area and negative correlations at CpG sites representing the gene body.

Of note, we found both TIM-3 and LGALS9 mRNA expression and DNA methylation correlating differently in identical cell types depending on their differentiation state. The activated state correlated positively with mRNA expression and gene body methylation (and negatively with promoter methylation), while the naïve or resting state correlated negatively (and positively with promoter methylation), respectively. We observed this for M0 and M1 macrophages, activated and resting NK cells, as well as activated and resting memory/naïve CD4^+^ T cells (Fig. 5, Additional file 1: Table S1).

However, it needs to be noted that not all beads showed significant correlations as already seen in analyses with the lymphocyte score and the IFN-γ signature. The most consistent correlations of methylation are seen at CpG site targeted by bead 6 for *HAVCR2* and at CpG site targeted by bead 12 for *LGALS9.*

### TIM-3 and LGAL9S mRNA expression is strongly associated with survival

Finally, we investigated the association of mRNA expression and methylation with patient overall survival. Log2-transformed mRNA expression levels of TIM-3 and LGALS9 showed a significant association with beneficial survival (Table 1). However, continuous methylation levels at all analyzed loci failed to reach statistical significance regarding their association with overall survival.

**Fig. 6:**
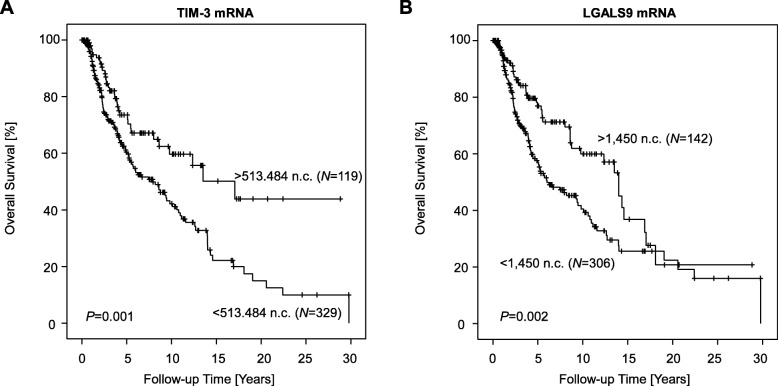
Kaplan–Meier analysis of overall survival in melanoma patients stratified according to *TIM*-*3* (**a**) and *LGALS9* (**b**) mRNA expression. Patient samples were dichotomized based on optimized cut-offs in *N* = 468 melanoma patients from The Cancer Genome Atlas. *P* values refer to the log rank test

We further investigated survival differences in groups of patients categorized as mRNA_high_ and mRNA_low_, respectively. We dichotomized mRNA expression levels using an optimized cut-off. The optimal cut-off for mRNA expression was 513.484 n.c. for TIM-3 and 1450 n.c. for LGALS9. Patients with high expression of TIM-3 and LGAL9S had significantly better overall survival compared to patients with low expressing tumors (Fig. 6). Patients whose tumors express TIM-3 mRNA above the cut-off had a median overall survival of 17.1 years as compared to 7.9 years for patients whose tumors express TIM-3 mRNA below the cut-off (*P* = 0.001). Patients with LGALS9 mRNA-high tumors showed a median survival of 14.0 years as compared to only 6.0 years for patients with LGALS9 mRNA-low tumors (*P* = 0.002).

## Discussion

In the present study, we provide a comprehensive overview of DNA methylation of *TIM*-*3* and its ligand *LGALS9* in melanomas, melanocytes, and immune cells. Firstly, for both TIM-3 and LGALS9, we detected significant positive correlations between mRNA expression and gene body methylation and inverse correlations between mRNA expression with promoter methylation, suggesting that DNA methylation could epigenetically regulate both genes. Secondly, TIM-3 and LGALS9 mRNA expression and methylation levels correlated significantly with tumor immune cell infiltration. For the assessment of the tumor infiltrates, we used different measurements such as lymphocyte score, tumor purity, leukocyte fraction, and RNA signatures of immune cell subsets. Thirdly, we compared the methylation status of *HAVCR2*/*TIM*-*3* and *LGALS9* with isolated monocytes, granulocytes, B cells, CD8^+^ T cells, and CD4^+^ T cells from healthy donors and melanocyte and melanoma cell lines. Here, we observed significant differences of methylation levels between the immune cells, melanocytes, and melanoma cells, which was most pronounced for the CpG site targeted by bead 6 (*HAVCR2*) and bead 12 (*LGALS9*). Strong, significant correlations targeting these two beads were consistent throughout our analyses. Since mRNA expression data was not available for isolated blood cells, correlations between methylation and mRNA expression need to be analyzed in isolated immune cell populations in future studies. Finally, we found high TIM-3 and LGALS9 mRNA expression to be associated with a significant better overall survival.

Expression of TIM-3 has been shown to be enriched in the tumor microenvironment compared to blood or lymphatic tissue, and its expression is dependent on IFN-γ [7]. Concordantly, we found positive correlations between TIM-3/LGALS9 mRNA expression and the IFN-γ signature. As expected, we observed a correlation between methylation and the IFN-γ signature consistent with the methylation pattern that correlates positively with mRNA expression. Strong positive correlations were observed between mRNA expression and activated T cells, natural killer cells, dendritic cells, macrophages and, in contrast, negative correlations for the respective resting or naïve state cell types. Altogether, these results support that DNA methylation plays a role in regulating expression of *TIM*-*3* and its ligand *LGALS9*. DNA methylation of those genes could thus serve as a surrogate biomarker for tumor infiltration by TIM-3- and LGALS9-expressing immune cells.

Despite the great achievements with current ICB, response rates vary greatly between patients. While some patients show profound and long-lasting responses, others do not respond at all [32]. Multiple clinical trials with various anti-TIM-3 antibodies (BMS-986258 (Bristol-Myers Squibb, New York City, New York, United States), TSR-022 (Tesaro, Waltham, Massachusetts, United States), LY3321367, and LY3415244 (Eli Lilly and Company, Indianapolis, Indiana, United States); INCAGN02390 (Incyte, Wilmington, Delaware, United States), MGB453 (Novartis, Basel, Switzerland), Sym023 (Symphogen A/S, Copenhagen, Denmark), RO7121661 (Hoffmann-La Roche, Basel, Switzerland), BGB-A425 (BeiGene, Peking, China)) are currently ongoing (ClinicalTrials.gov Identifiers: NCT03489343, NCT03680508, NCT02817633, NCT03099109, NCT02608268, NCT03652077, NCT03066648, NCT03446040, NCT03708328, NCT03311412, NCT03744468, NCT03752177, NCT03940352, NCT03307785). Initial results of these studies are expected in the near future. However, it can be speculated that, similar to other ICB treatments, not all patients will respond to TIM-3 blockade. Hence, it is of eminent importance to stratify patients to identify those that are most likely to benefit from specific ICB treatments. Therefore, a focus of recent research in this field is the identification of predictive biomarkers for responders versus non-responders. Biomarkers that were found to be inadequate in predicting the outcome of ICB treatment in melanoma include blood levels of S100 and LDH, tumor-infiltrating lymphocytes, or the immuno-score, genomic stability, and the mutational burden [9]. The most promising candidate, PD-L1 expression assessed by immunohistochemistry, has also shown mixed results and the importance of soluble PD-L1 as a predictive biomarker for ICB in melanoma remains to be validated [9, 33, 34]. However, recently, Goltz et al. provided data suggesting that DNA methylation of the immune checkpoint gene *CTLA4* predicts response to CTLA-4- and PD-1-targeted ICB [35]. Our results suggest that methylation testing of *TIM*-*3* could be a promising predictive biomarker to select the subset of melanoma patients who would benefit from TIM-3-targeted ICB. Accordingly, future studies should address the value of *TIM*-*3* methylation testing as companion biomarker for TIM-3 ICB.

Factors for treatment resistance during ICB include insufficient anti-tumor T cell generation, impaired formation of T cell memory, and T cell exhaustion [36, 37]. Exhausted T cells are dysfunctional T cells with a state-specific epigenetic landscape [38–40]. T cell exhaustion is a progressive process occurring during continuous T cell stimulation, accompanied by a progressive loss of effector function [7, 37, 41]. Upregulation of immune checkpoint molecules, such as TIM-3, PD-1, or LAG3, is a key feature of T cell exhaustion [37, 38, 42]. Prior studies described hypomethylation of TIM-3 being associated with T cell exhaustion [43]. Our study showed that TIM-3 promoter methylation correlated inversely with mRNA expression. It would be interesting to carry out methylation analysis of isolated, exhausted T cells, in order to examine if hypomethylation of CpG sites located in the TIM-3 promoter area might serve as a surrogate biomarker for T cell exhaustion.

While ICB can lead to rejuvenation of exhausted T cells and facilitate an enhanced anti-tumor response, a portion of exhausted T cells cannot be re-invigorated [37, 38]. It has been suggested that T cells that co-express TIM-3 and PD-1 are functionally more impaired than T cells expressing either TIM-3 or PD-1 alone [42, 44]. TIM-3 and PD-1 are co-expressed in exhausted tumor-infiltrating T cells in melanoma patients [7, 45, 46]. Additionally, TIM-3 expression in melanoma cells is associated with non-responsiveness to PD-1 ICB and after PD-1-targeted ICB, upregulation of TIM-3 has been observed [44, 47, 48]. Transcriptional control of TIM-3 expression via DNA methylation suggests that DNA methylation of immune checkpoint genes could be involved in the development of ICB resistance.

The differentiation of naïve T cells into effector T cells, and eventually exhausted T cells, involves changes in DNA methylation, including changes of the methylation status of immune checkpoints [41, 49–51]. Data recently published by Ghoneim et al. suggests that de novo methylation plays a critical role in terminal T cell exhaustion, a stable epigenetic state persisting after PD-1 ICB and resulting in PD-1 ICB failure [41]. In the context of immunotherapy, aberrant DNA methylation in melanoma has been described in multiple studies. For example, Chatterjee et al. showed that DNA methylation influences PD-L1 expression in melanoma cells, with hypomethylation being accompanied by PD-L1 upregulation [43, 52]. Concordant results were found by Micevic and colleagues, who analyzed the effect of pharmaceutical demethylation on PD-L1 expression [53]. Interestingly, treatment with DNA methyltransferase inhibitors has also been shown to trigger immune response in different cancer types [54]. Combinational approaches of hypomethylating agents with immunotherapy are ongoing, and in that context, DNA methylation testing could also serve as a predictive biomarker (reviewed in [25]).

Currently, predictive biomarkers include gene expression analysis of immune checkpoints, tumor mutational load, and the intensity of CD8^+^ TILs [55]. DNA methylation is an attractive biomarker since it can be accurately quantified even in formalin-fixed and paraffin-embedded tissues and is biologically and chemically more stable than gene and protein expression [24]. Accordingly, previous studies have shown DNA methylation of various genes to be valid prognostic biomarkers in different cancers [56–60]. Additionally, Nair et al. have already shown that epigenetic modification of TIM-3 in human colorectal and breast cancer could be a useful biomarker in these diseases [27, 28]. In the future, methylation testing of TIM-3 might serve as a predictive biomarker for melanoma patients.

We are aware of the limitations of our study. Firstly, we used different immune signatures as surrogates for distinct tumor-infiltrating leukocytes. Since TIM-3 can be expressed on various different cell types in the tumor microenvironment, including effector T cells, cells of the innate immune system, and melanoma cells [7, 8], a detailed analysis of isolated pure cell populations from melanomas is required. In addition, further studies with in-depth analyses of methylation changes during T cell exhaustion are warranted. These would also help elucidate whether the differences in methylation state of *TIM*-*3* and *LGALS9* in the present study distinguish “immunologically hot” tumors with high immune cell infiltration from poorly infiltrated, “immunologically cold,” tumors [61].

Secondly, we found that TIM-3 and LGALS9 mRNA expression correlated with beneficial overall survival; however, methylation analysis failed to accompany this finding. Given that TIM-3 expression is typically associated with T cell exhaustion, and exhausted T cells fail to effectively suppress cancer cells, one may expect a worse survival outcome in high TIM-3-expressing tumors. In accordance with this hypothesis, multiple recent studies found that high levels of TIM-3 expression in tumors were associated with worse overall survival (reviewed in [42]). However, in our analysis, we found opposing results for melanoma, as high TIM-3 mRNA expression correlated with better overall survival. However, we also observed that TIM-3 mRNA expression correlated with tumor immune cell infiltration. Although high TIM-3 expression may suggest greater T cell exhaustion, a significant portion of leukocytes might still be in the effector phase in “immunologically hot” tumors. Therefore, the better survival of patients with high TIM-3 and LGALS9 expression observed in our study might be due to a better immune response in the tumor, regardless of the high expression of TIM-3. Consistent with this hypothesis, studies have found that patients with highly immunologically infiltrated melanoma had significantly better outcomes compared to those with low immune infiltration [29, 62].

Thirdly, we analyzed in total six CpG sites for *HAVCR2* and eight for *LGALS9* rather than sampling all CpG sites located in that area. Our results show differences between the CpG sites depending on the localization on the gene. An analysis of the total CpG sites in that area might provide additional information about potential correlations. Unfortunately, the Illumina HumanMethylation450 BeadChip as used by the TCGA Research Network does not cover all CpG sites within the region of interest. Methods like bisulfite sequencing would be suitable, and further analysis should be performed to provide a deeper insight at single CpG site resolution. Nevertheless, a strength of our study is the high number of analyzed patient samples provided by the TCGA Research Network. The large patient collective resulted in a high number of statistically significant results, even though correlations were weak at multiple CpG sites.

Finally, our analysis showed significant correlations between *HAVCR2*/*TIM*-*3* methylation status (in both the promoter area and gene body) and *BRAF*-mutational subtype in melanoma. Goltz et al. previously showed that promoter methylation of *CTLA4* correlates with *BRAF* mutational status in melanoma [35]. Frederick et al. detected changes in the tumor microenvironment under BRAF inhibition with an increase of cytotoxic CD8^+^ T infiltrates and enhanced cytotoxic markers. Interestingly, TIM-3 expression, which was used as surrogate for T cell exhaustion under treatment, was also increased [63]. Several other studies using melanoma cell lines found augmented anti-tumor immune responses under BRAF inhibition [64–66]. Murine models have already shown the potential of the combination of ICB and BRAF inhibition for an enhanced therapy response [67]. With regard to combinational ICB and BRAF targeted therapies, it would be interesting to investigate whether methylation status of immune checkpoint molecules such as TIM-3 can serve as predictive biomarkers. Altogether, this might provide rationale for future studies investigating potential pathophysiologic connections between immune checkpoint expression, among them TIM-3, and *BRAF* mutations, or BRAF inhibition in melanoma.

## Materials and methods

The aim of the study was the investigation of DNA methylation status and the corresponding mRNA levels of TIM-3 and its ligand LGALS9 in melanoma patients that were provided by the Cancer Genome Atlas. Moreover, we evaluated DNA methylation levels in melanoma and melanocyte cell lines, as well as isolated immune cells from healthy donors. We furthermore aimed to examine potential associations between DNA methylation/mRNA expression of the respective genes and clinical-pathological parameters, molecular and immunologic features, and patient’s survival.

### Patient samples and ethics

We used data provided by TCGA Research Network for our analysis (http://cancergenome.nih.gov). We included data from *N* = 470 samples of the TCGA skin cutaneous melanoma (SKCM) cohort. One sample per patient was analyzed, including primary, lymph node, and metastatic tissue. For patients providing primary tumor as well as metastatic tumor tissue, the primary tumor tissue sample was used. Information about clinical-pathological and molecular data, such as RNAseq data or methylation analysis, was obtained from the previously published TCGA Research Network [29] and is listed in Additional file 1: Table S1. Information about tumor purity and ploidy was transferred from the TCGA Research Network and calculated using the ABSOLUTE algorithm [68].

For the analyses of tumor immune cell infiltrates, we again exploited data of the TCGA Research Network including lymphocyte distribution, lymphocyte density, and lymphocyte score, which is derived from the density and distribution of melanoma-associated lymphocytes and calculated as described (Additional file 1: Table S1) [29]. As a surrogate measure for the immune infiltration, we used the tumor purity. Tumor purity described the contamination of a tumor with non-tumor cells that do not carry tumor-specific mutations, and is at least partly determined by the immune infiltration [29, 68]. We further included quantitative data on immune signatures provided by Thorsson et al. [30] and additionally the tumor-infiltrating leukocyte fraction quantified based on DNA methylation arrays provided by Saltz et al. [31] (Additional file 1 Table S1). Finally, we included DNA methylation data from human melanoma (*N* = 9) and melanocyte (*N* = 3) cell lines (Gene Expression Omnibus (GEO) accessions: GSE51547, GSE44662), and from isolated leukocytes (monocytes, granulocytes, B cells, CD8^+^ T cells, CD4^+^ T cells) derived from peripheral blood of healthy patients (*N* = 28, GSE103541). For blood leukocytes and cell lines, no mRNA expression data was available.

The TCGA Research Network obtained informed consent from all patients in accordance with the Declaration of Helsinki 1975.

### mRNA expression analysis

mRNA expression levels provided by TCGA were assessed via llumina HiSeq 2000 RNA Sequencing Version 2 analysis (Illumina, Inc., San Diego, CA, USA) and normalized counts (n.c.) per transcript were calculated with the SeqWare framework via the RNA-Seq by Expectation Maximization (RSEM) algorithm [69].

### Methylation analysis

DNA methylation levels were quantified using the Infinium HumanMethylation450 BeadChip (Illumina, Inc., San Diego, CA, USA) technology. In accordance with prior studies, methylation levels (beta values) were calculated as follows: beta-value = (Intensity_Methylated) / (Intensity_Methylated + Intensity_Unmethylated + α) [70]. The constant offset α was set to 0. To show methylation levels between 0 and 100%, beta values (between 0 and 1) were multiplied times 100%.

### Statistical analysis

To evaluate potential correlations between groups, we performed Spearman’s rank correlations (Spearman’s *ρ*). Comparisons between groups were conducted using Mann–Whitney *U* and Kruskal–Wallis tests. Overall survival was investigated via Kaplan–Meier and Cox proportional hazards analyses. *P* values refer to log-rank for Kaplan–Meier and Wald tests for Cox proportional analyses. Cox proportional hazards were calculated with log2-transformed methylation and mRNA expression data. Dichotomization of mRNA expression levels for Kaplan–Meier analyses was performed applying optimized cut-offs. The optimized cut-off was defined as the value which yielded the smallest *P* value (log-rank test) when comparing survival differences between both groups. For log2-transformation, mRNA expression levels of 0 n.c. were set to 0.1. *P* values < 0.05 were considered statistically significant.

## Supplementary information

**Additional file 1.** Associations and correlations of *TIM*-*3* and *LGALS9* methylation and mRNA expression with clinical-pathological parameters, molecular features and immune cell infiltrates in *N* = 470 melanoma patients from The Cancer Genome Atlas. Molecular data were obtained from The Cancer Genome Atlas [28]. *P*-values refer to Kruskal-Wallis (> two group comparisons), Wilcoxon Mann-Whitney *U* (2 group comparison) tests, and Spearman’s rank correlation (continuous variables), respectively.

## Data Availability

The dataset used and/or analyzed during the current study are available from the corresponding author on reasonable request, from the TCGA network, and from Gene Expression Omnibus (GEO; https://www.ncbi.nlm.nih.gov/geo/, GEO accessions: GSE51547, GSE44662, GSE103541).

## Abbreviations

95% CI: 95% Confidence interval
CTLA-4: Cytotoxic T-lymphocyte-associated protein 4
DC: Dendritic cells
FDA: Food and Drug Administration
GEO: Gene Expression Omnibus
*HAVCR2*: Hepatitis A virus cellular receptor 2
HR: Hazard ratio
ICB: Immune checkpoint blockade
IFN-γ: Interferon-γ
LAG-3: Lymphocyte-activation gene 3
LGALS9: Galectin 9
NK cells: Natural killer cells
PD-1: Programmed cell death protein 1
PDL-1: Programmed cell death ligand 1
RSEM: RNA-Seq by expectation maximization
SKCM: Skin cutaneous melanoma
TCGA: The Cancer Genome Atlas
Th1 cells: T helper 1 cells
TILs: Tumor-infiltrating lymphocytes
TIM-3: T cell immunoglobulin and mucin-domain containing-3 receptor

## References

[1] Kaiser J, Couzin-Frankel J (2018). Cancer immunotherapy sweeps Nobel for medicine. Science..

[2] Pardoll DM (2012). The blockade of immune checkpoints in cancer immunotherapy. Nat Rev Cancer.

[3] Hodi FS, Mihm MC, Soiffer RJ (2003). Biologic activity of cytotoxic T lymphocyte-associated antigen 4 antibody blockade in previously vaccinated metastatic melanoma and ovarian carcinoma patients. PNAS.

[4] Weber J, Mandala M, Del Vecchio M, Gogas HJ, Arance AM, Cowey CL (2017). Adjuvant Nivolumab versus Ipilimumab in resected stage III or IV melanoma. N Engl J Med.

[5] Robert C, Schachter J, Long GV, Arance A, Grob JJ, Mortier L (2015). Pembrolizumab versus Ipilimumab in advanced melanoma. N Engl J Med.

[6] Domingues B, Lopes JM, Soares P, Pópulo H (2018). Melanoma treatment in review. ITT..

[7] Anderson AC (2012). Tim-3, a negative regulator of anti-tumor immunity. Curr Opin Immunol.

[8] Anderson AC, Joller N, Kuchroo VK (2016). Lag-3, Tim-3, and TIGIT: co-inhibitory receptors with specialized functions in immune regulation. Immunity..

[9] Nishino M, Ramaiya NH, Hatabu H, Hodi FS (2017). Monitoring immune-checkpoint blockade: response evaluation and biomarker development. Nat Rev Clin Oncol.

[10] Fagerberg L, Hallström BM, Oksvold P, Kampf C, Djureinovic D, Odeberg J (2014). Analysis of the human tissue-specific expression by genome-wide integration of transcriptomics and antibody-based proteomics. Mol Cell Proteomics.

[11] Thijssen VL, Hulsmans S, Griffioen AW (2008). The galectin profile of the endothelium: altered expression and localization in activated and tumor endothelial cells. Am J Pathol.

[12] Wada J, Ota K, Kumar A, Wallner EI, Kanwar YS (1997). Developmental regulation, expression, and apoptotic potential of galectin-9, a beta-galactoside binding lectin. J Clin Invest.

[13] Lai JH, Luo SF, Wang MY, Ho LJ. Translational implication of galectin-9 in the pathogenesis and treatment of viral infection. Int J Mol Sci. 2017;18(10) 10.3390/ijms18102108 PMC5666790 28991189

[14] Bi S, Earl LA, Jacobs L, Baum LG (2008). Structural features of galectin-9 and galectin-1 that determine distinct T cell death pathways. J Biol Chem.

[15] Spitzenberger F, Graessler J, Schroeder HE (2001). Molecular and functional characterization of galectin 9 mRNA isoforms in porcine and human cells and tissues. Biochimie..

[16] Chou FC, Chen HY, Kuo CC, Sytwu HK. Role of galectins in tumors and in clinical immunotherapy. Int J Mol Sci. 2018;19(2) 10.3390/ijms19020430 PMC5855652 29389859

[17] Zhu C, Anderson AC, Schubart A, Xiong H, Imitola J, Khoury SJ (2005). The Tim-3 ligand galectin-9 negatively regulates T helper type 1 immunity. Nat Immunol.

[18] Monney L, Sabatos CA, Gaglia JL, Ryu A, Waldner H, Chernova T (2002). Th1-specific cell surface protein Tim-3 regulates macrophage activation and severity of an autoimmune disease. Nature..

[19] Meyers JH, Sabatos CA, Chakravarti S, Kuchroo VK (2005). The TIM gene family regulates autoimmune and allergic diseases. Trends Mol Med.

[20] Yang R, Hung M-C (2017). The role of T-cell immunoglobulin mucin-3 and its ligand galectin-9 in antitumor immunity and cancer immunotherapy. Sci China Life Sci.

[21] Taby R, Issa JP (2010). Cancer epigenetics. CA Cancer J Clin.

[22] Schmidl C, Delacher M, Huehn J, Feuerer M (2018). Epigenetic mechanisms regulating T-cell responses. J Allergy Clin Immunol.

[23] Ghoneim HE, Zamora AE, Thomas PG, Youngblood BA (2016). Cell-intrinsic barriers of T cell-based immunotherapy. Trends Mol Med.

[24] Jones PA (2012). Functions of DNA methylation: islands, start sites, gene bodies and beyond. Nat Rev Genet.

[25] Micevic G, Theodosakis N, Bosenberg M (2017). Aberrant DNA methylation in melanoma: biomarker and therapeutic opportunities. Clin Epigenetics.

[26] Chou FC, Kuo CC, Chen HY, Chen HH, Sytwu HK (2016). DNA demethylation of the TIM-3 promoter is critical for its stable expression on T cells. Genes Immun.

[27] Sasidharan Nair V, Toor SM, Taha RZ, Shaath H, Elkord E (2018). DNA methylation and repressive histones in the promoters of PD-1, CTLA-4, TIM-3, LAG-3, TIGIT, PD-L1, and galectin-9 genes in human colorectal cancer. Clin Epigenetics.

[28] Sasidharan Nair V, El Salhat H, Taha RZ, John A, Ali BR, Elkord E (2018). DNA methylation and repressive H3K9 and H3K27 trimethylation in the promoter regions of PD-1, CTLA-4, TIM-3, LAG-3, TIGIT, and PD-L1 genes in human primary breast cancer. Clin Epigenetics.

[29] Network CGA (2015). Genomic classification of cutaneous melanoma. Cell..

[30] Thorsson V, Gibbs DL, Brown SD, Wolf D, Bortone DS, Ou Yang TH (2018). The immune landscape of cancer. Immunity.

[31] Saltz J, Gupta R, Hou L, Kurc T, Singh P, Nguyen V (2018). Spatial organization and molecular correlation of tumor-infiltrating lymphocytes using deep learning on pathology images. Cell Rep.

[32] Schadendorf D, van Akkooi ACJ, Berking C, Griewank KG, Gutzmer R, Hauschild A (2018). Melanoma Lancet.

[33] Khunger M, Hernandez AV, Pasupuleti V, Rakshit S, Pennell NA, et al. Programmed cell death 1 (PD-1) ligand (PD-L1) expression in solid tumors as a predictive biomarker of benefit from PD-1/PD-L1 axis inhibitors: a systematic review and meta-analysis. JCO Prec Oncol. 2017; 10.1200/PO.16.00030. JCO Precision Oncology - published online May 18, 2017 10.1200/PO.16.00030 35172490

[34] Zhou J, Mahoney KM, Giobbie-Hurder A, Zhao F, Lee S, Liao X (2017). Soluble PD-L1 as a biomarker in malignant melanoma treated with checkpoint blockade. Cancer Immunol Res.

[35] Goltz D, Gevensleben H, Vogt TJ, Dietrich J, Golletz C, Bootz F, et al. CTLA4 methylation predicts response to anti-PD-1 and anti-CTLA-4 immunotherapy in melanoma patients. JCI Insight. 2018;3(13) 10.1172/jci.insight.96793 PMC6124533 29997292

[36] Jenkins RW, Barbie DA, Flaherty KT (2018). Mechanisms of resistance to immune checkpoint inhibitors. Br J Cancer.

[37] Catakovic K, Klieser E, Neureiter D, Geisberger R (2017). T cell exhaustion: from pathophysiological basics to tumor immunotherapy. Cell Commun Signal.

[38] Makoto Kurachi. CD8+ T cell exhaustion. Semin Immunol 2019. Accessed from: https://www.springermedizin.de/cd8-t-cell-exhaustion/16656592. Accessed May 2019.

[39] Sen DR, Kaminski J, Barnitz RA, Kurachi M, Gerdemann U, Yates KB (2016). The epigenetic landscape of T cell exhaustion. Science..

[40] Pauken KE, Sammons MA, Odorizzi PM, Manne S, Godec J, Khan O (2016). Epigenetic stability of exhausted T cells limits durability of reinvigoration by PD-1 blockade. Science..

[41] Ghoneim HE, Fan Y, Moustaki A, Abdelsamed HA, Dash P, Dogra P (2017). De novo epigenetic programs inhibit PD-1 blockade-mediated T cell rejuvenation. Cell.

[42] Das M, Zhu C, Kuchroo VK (2017). Tim-3 and its role in regulating anti-tumor immunity. Immunol Rev.

[43] Emran AA, Chatterjee A, Rodger EJ, Tiffen JC, Gallagher SJ, Eccles MR (2019). Targeting DNA methylation and EZH2 activity to overcome melanoma resistance to immunotherapy. Trends Immunol.

[44] Sakuishi K, Apetoh L, Sullivan JM, Blazar BR, Kuchroo VK, Anderson AC (2010). Targeting Tim-3 and PD-1 pathways to reverse T cell exhaustion and restore anti-tumor immunity. J Exp Med.

[45] Fourcade J, Sun Z, Benallaoua M, Guillaume P, Luescher IF, Sander C (2010). Upregulation of Tim-3 and PD-1 expression is associated with tumor antigen-specific CD8+ T cell dysfunction in melanoma patients. J Exp Med.

[46] Baitsch L, Baumgaertner P, Devêvre E, Raghav SK, Legat A, Barba L, Wieckowski S (2011). Exhaustion of tumor-specific CD8^+^ T cells in metastases from melanoma patients. J Clin Invest.

[47] Keenan TE, Burke KP, Van Allen EM (2019). Genomic correlates of response to immune checkpoint blockade. Nat Med.

[48] Granier C, Dariane C, Combe P, Verkarre V, Urien S, Badoual C (2017). Tim-3 expression on tumor-infiltrating PD-1+CD8+ T cells correlates with poor clinical outcome in renal cell carcinoma. Cancer Res.

[49] Durek P, Nordström K, Gasparoni G, Salhab A, Kressler C, de Almeida M (2016). Epigenomic profiling of human CD4+ T cells supports a linear differentiation model and highlights molecular regulators of memory development. Immunity..

[50] Scharer CD, Barwick BG, Youngblood BA, Ahmed R, Boss JM (2013). Global DNA methylation remodeling accompanies CD8 T cell effector function. J Immunol.

[51] Ahn E, Youngblood B, Lee J, Lee J, Sarkar S, Ahmed R (2016). Demethylation of the PD-1 promoter is imprinted during the effector phase of CD8 T cell exhaustion. J Virol.

[52] Chatterjee A, Rodger EJ, Ahn A, Stockwell PA, Parry M, Motwani J (2018). Marked global DNA hypomethylation is associated with constitutive PD-L1 expression in melanoma. iScience.

[53] Micevic G, Thakral D, McGeary M, Bosenberg MW (2019). PD-L1 methylation regulates PD-L1 expression and is associated with melanoma survival. Pigment Cell Melanoma Res.

[54] Chiappinelli KB, Strissel PL, Desrichard A, Li H, Henke C, Akman B (2017). Inhibiting DNA methylation causes an interferon response in Cancer via dsRNA including endogenous retroviruses. Cell..

[55] Topalian SL, Taube JM, Anders RA, Pardoll DM (2016). Mechanism-driven biomarkers to guide immune checkpoint blockade in cancer therapy. Nat Rev Cancer.

[56] Esteller M, Garcia-Foncillas J, Andion E, Goodman SN, Hidalgo OF, Vanaclocha V (2000). Inactivation of the DNA-repair gene MGMT and the clinical response of gliomas to alkylating agents. N Engl J Med.

[57] Church TR, Wandell M, Lofton-Day C, Mongin SJ, Burger M, Payne SR (2014). Prospective evaluation of methylated SEPT9 in plasma for detection of asymptomatic colorectal cancer. Gut..

[58] Partin AW, Van Neste L, Klein EA, Marks LS, Gee JR, Troyer DA (2014). Clinical validation of an epigenetic assay to predict negative histopathological results in repeat prostate biopsies. J Urol.

[59] van Kessel KE, Beukers W, Lurkin I, Ziel-van der Made A, van der Keur KA, Boormans JL (2017). Validation of a DNA methylation-mutation urine assay to select patients with hematuria for cystoscopy. J Urol.

[60] Imperiale TF, Ransohoff DF, Itzkowitz SH (2014). Multitarget stool DNA testing for colorectal-cancer screening. N Engl J Med.

[61] Popovic A, Jaffee EM, Zaidi N (2018). Emerging strategies for combination checkpoint modulators in cancer immunotherapy. J Clin Invest.

[62] Azimi F, Scolyer RA, Rumcheva P, Moncrieff M, Murali R, McCarthy SW (2012). Tumor-infiltrating lymphocyte grade is an independent predictor of sentinel lymph node status and survival in patients with cutaneous melanoma. J Clin Oncol.

[63] Frederick DT, Piris A, Cogdill AP, Cooper ZA, Lezcano C, Ferrone CR (2013). BRAF inhibition is associated with enhanced melanoma antigen expression and a more favorable tumor microenvironment in patients with metastatic melanoma. Clin Cancer Res.

[64] Boni A, Cogdill AP, Dang P, Udayakumar D, Njauw CN, Sloss CM (2010). Selective BRAFV600E inhibition enhances T-cell recognition of melanoma without affecting lymphocyte function. Cancer Res.

[65] Khalili JS, Liu S, Rodríguez-Cruz TG, Whittington M, Wardell S, Liu C (2012). Oncogenic BRAF(V600E) promotes stromal cell-mediated immunosuppression via induction of interleukin-1 in melanoma. Clin Cancer Res.

[66] Liu C, Peng W, Xu C, Lou Y, Zhang M, Wargo JA (2013). BRAF inhibition increases tumor infiltration by T cells and enhances the antitumor activity of adoptive immunotherapy in mice. Clin Cancer Res.

[67] Cooper ZA, Juneja VR, Sage PT, Frederick DT, Piris A, Mitra D (2014). Response to BRAF inhibition in melanoma is enhanced when combined with immune checkpoint blockade. Cancer Immunol Res..

[68] Carter SL, Cibulskis K, Helman E, McKenna A, Shen H, Zack T (2012). Absolute quantification of somatic DNA alterations in human cancer. Nat Biotechnol.

[69] Li B, Dewey CN (2011). RSEM: accurate transcript quantification from RNA-Seq data with or without a reference genome. BMC Bioinformatics.

[70] Du P, Zhang X, Huang CC, Jafari N, Kibbe WA, Hou L (2010). Comparison of beta-value and M-value methods for quantifying methylation levels by microarray analysis. BMC Bioinformatics..

